# Supplementary material for: Lipid ROS- and Iron-Dependent Ferroptotic Cell Death in Unicellular Algae Chlamydomonas reinhardtii
Source: Cells. 2023 Feb 8;12(4):553. doi: 10.3390/cells12040553 (PMC9953829; doi:10.3390/cells12040553)
Supplement: Supplementary file 1 [file cells-12-00553-s001.zip › cells-2115654-supplementary.pdf]

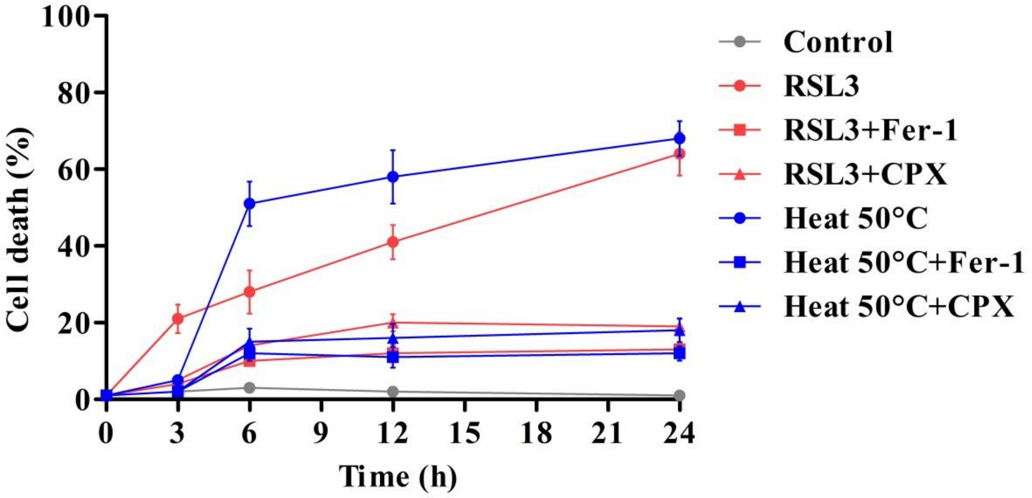

Figure S1. Cell death kinetics of *Chlamydomonas* cells subjected to RSL3 and heat (50 °C) treatment with or without ferroptosis inhibitors. The data are represented as mean ± SD (n=3).

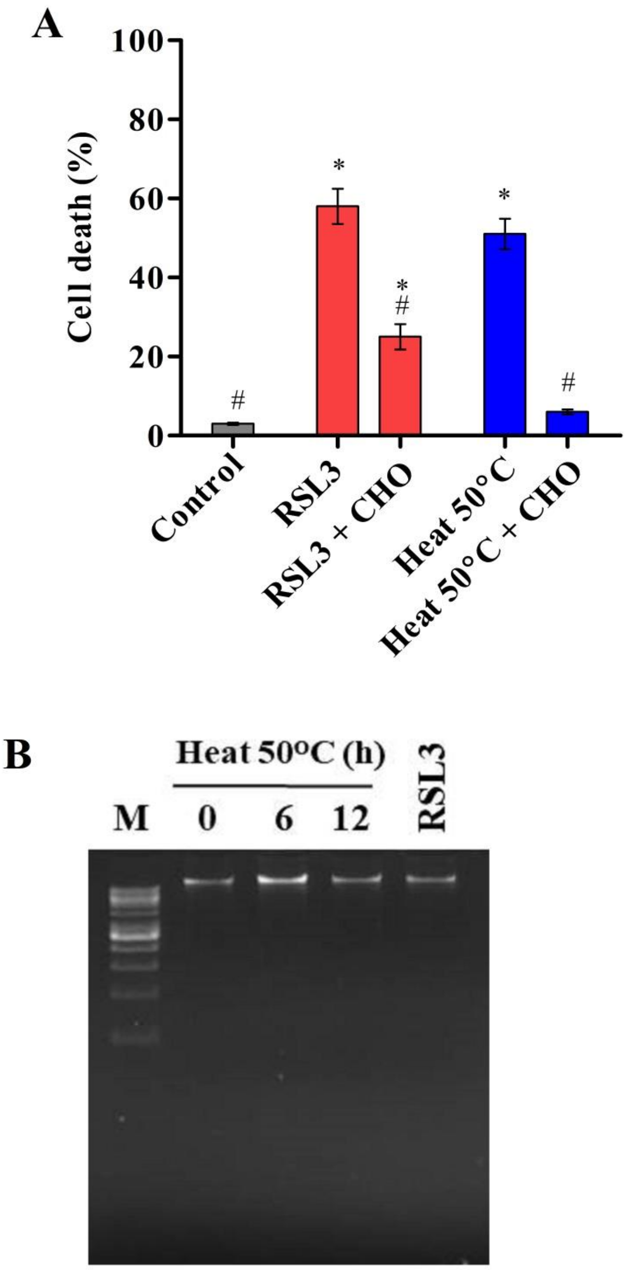

Figure S2. The effect of heat shock and RSL3 treatment on caspase-like activity (A) and DNA fragmentation (B) in *Chlamydomonas* cells. 10kb DNA marker (M) was used. The data are represented as mean ± SD (n=3). * indicates significant differences with respect to untreated control. # indicates significant differences with respect to RSL3 or Heat 50ºC treatment ($p<0.05$)

Table S1. List of RT-qPCR primers used in this study

Primers	Sequence (5′-3′)	Length (bp)	Annealing Temperature (°C)
GPX5-F	GCGGTCGCCAATAACCAAT	19	60
GPX5-R	AAGGGCTGTCCCGAAAGC	18	
HO-1-F	CTGCCGACATTGCCTGGAT	18	60
HO-1-R	CGCCTCTTGCTTTATGCCTC	20	
CBLP-F	CTTCTCGCCCATGACCAC	18	60
CBLP-R	CCCACCAGGTTGTTCTTCAG	20	
